# Protrieve Sheath embolic protection during venous thrombectomy: early experience in seventeen patients

**DOI:** 10.1186/s42155-024-00484-0

**Published:** 2024-10-09

**Authors:** Colvin Greenberg, David S. Shin, Luke Verst, Eric J. Monroe, Frederic J. Bertino, Matthew Abad-Santos, Jeffrey Forris Beecham Chick

**Affiliations:** 1 Department of Radiology, Section of Vascular and Interventional Radiology, University of Washington, 1959 Northeast Pacific Street, Seattle, WA 98195 USA; 2 Department of Radiology, Division of Vascular and Interventional Radiology, University of Southern California, 1500 San Pablo Street, Los Angeles, CA 90033 USA; 3 Department of Radiology, University of Wisconsin, 1675 Highland Ave, Madison, WI 53792 USA; 4 Department of Radiology, NYU Langone Health/NYU Grossman School of Medicine, Tisch Hospital 2, Floor, 550 First Avenue, New York, NY 10016 USA

**Keywords:** Protrieve Sheath, Embolic protection, Venous thromboembolism, Cardioembolic events, Venous thrombectomy, Venous disease

## Abstract

**Purpose:**

The Protrieve Sheath (Inari Medical; Irvine, CA) is designed for embolic protection during venous thrombectomy. This report describes experience with its use.

**Materials and methods:**

Between November 2022 and December 2023 (13 months), seventeen patients, including nine (52.9%) females and eight (47.1%) males (mean age 58.8 ± 13.3 years, range 37–81 years), underwent deep venous thrombectomy following the Protrieve Sheath placement for embolic protection. Gender, age, presenting symptoms, procedural indications, obstructed venous segments, the Protrieve Sheath access and deployment sites, thrombectomy devices utilized, need for stent reconstruction, technical success, clinical success, adverse events (the Protrieve Sheath maldeployment or clinically significant embolic events), removed thrombi analyses, and mortality were recorded. Technical success was defined as successful deployment of the Protrieve Sheath funnel central to the thrombectomy site. Clinical success was defined as improvement in presenting venous occlusive symptoms without procedure-related venous thromboembolism.

**Results:**

The most common presenting symptom was extremity swelling (*n* = 15; 88.2%). Nine (52.9%) patients had malignant and eight (47.1%) had benign etiologies of venous obstruction. Obstructed venous segments included the inferior vena cava (IVC) and lower extremity (*n* = 9; 52.9%), isolated lower extremity (*n* = 4; 23.5%), isolated IVC (*n* = 2; 11.8%), thoracic central veins and superior vena cava (*n* = 1; 5.9%), and isolated thoracic central vein (*n* = 1; 5.9%). The Protrieve Sheath access sites included the right internal jugular vein (*n* = 15; 88.2%) for IVC and lower extremity obstructions and the right common femoral vein (*n* = 2; 11.8%) for thoracic central vein and superior vena cava obstructions. The Protrieve sheath funnel deployment locations included intrahepatic IVC in 13 patients (*n* = 13; 76.5%), suprarenal IVC in two (*n* = 2; 11.8%), and inferior cavoatrial junction in two (*n* = 2; 11.8%). Thrombectomy devices used included the ClotTriever System (Inari Medical) (*n* = 15; 88.2%), the InThrill Thrombectomy System (Inari Medical) (*n* = 4; 23.5%), the FlowTriever System (Inari Medical) (*n* = 2; 11.8%), the Lightning Flash 16 Aspiration System (Penumbra; Salt Lake City, UT) (*n* = 2; 11.8%), the Cleaner Rotational Thrombectomy System (Argon; Plano, TX) (*n* = 1; 5.9%), and the RevCore Thrombectomy System (Inari Medical) (*n* = 1; 5.9%). Ten (58.8%) patients required stent reconstruction following thrombectomy. Technical success was achieved in all patients. Clinical success was achieved in 16 (94.1%) patients. No immediate adverse events, including the Protrieve Sheath maldeployment or clinically significant embolic events, occurred.

**Conclusion:**

Use of the Protrieve Sheath during large-bore venous mechanical thrombectomy resulted in favorable technical and clinical outcomes without device-related adverse events or clinically significant thromboembolic events.

## Introduction

Embolic protection during endovascular procedures has long been considered, especially when treating atherosclerotic disease [1, 2]. Venous thromboembolism is a known possible adverse event during mechanical and pharmacomechanical thrombectomy [3, 4]. Since the advent of large-bore endovenous interventions, including mechanical thrombectomy and complex inferior vena cava (IVC) filter removal, there has been a growing interest in embolic protection in the central venous system. While temporary IVC filter placement is an option for intraprocedural embolic protection, the presence of the filter often hinders concurrent use of large-bore thrombectomy devices.

The Protrieve Sheath (Inari Medical; Irvine, CA) is designed to provide embolic protection during deep venous recanalization procedures. It consists of a 26-French outer diameter, 20-French inner diameter, 32-cm working length sheath. Its distal tip features a retractable self-expanding nitinol mesh funnel that opens to a maximum diameter of 33.5-mm and allows for circumferential wall apposition within the IVC. The large-bore sheath design facilitates the coaxial use of other catheter-based devices for thrombectomy, angioplasty, through-and-through access, or stent placement, without losing the protection during the intervention. The funnel traps any embolic materials that can be brought into the sheath and aspirated out the large-bore side-arm.

Limited case reports have described the utility of the Protrieve Sheath in capturing emboli during benign and malignant deep vein thrombectomy and IVC filter removal [5–8]. This study reports its feasibility and safety in a larger number of patients.

## Methods and materials

### Patients

*Patient demographic data is summarized in *Table 1. Seventeen patients, including nine (53%) females and eight (47%) males, with mean age of 58.8 ± 13.3 years (range: 37–81 years), underwent venous thrombectomy while using the Protrieve Sheath for embolic protection between November 2022 and December 2023 (13 months) at a tertiary academic medical center.

**Table 1:**
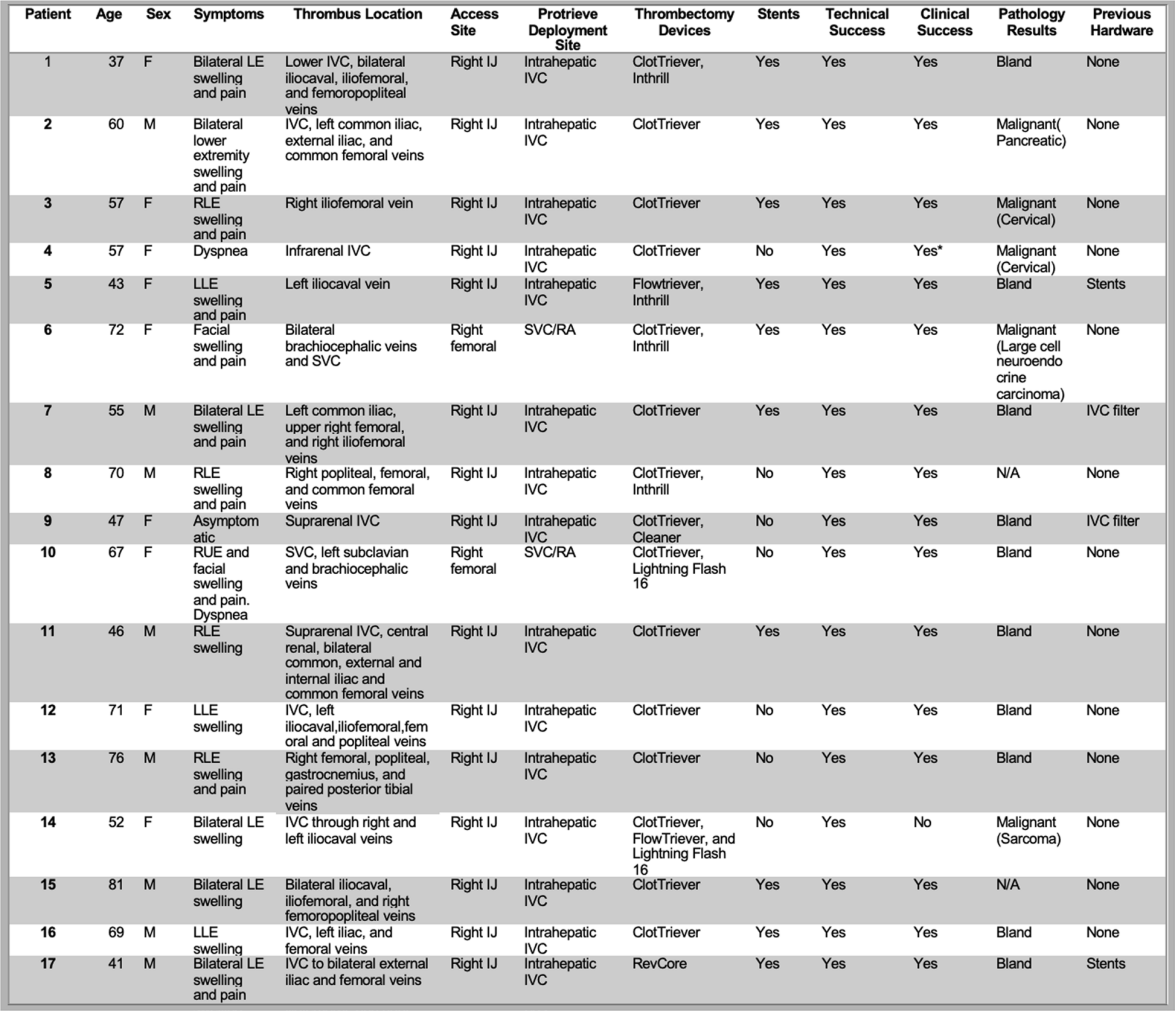
Patient demographic and results data

*LE* Lower extremity, *RLE* Right lower extremity, *LLE* Left lower extremity, *IVC* Inferior vena cava,* SVC* Superior vena cava,* IJ* Internal jugular

### Clinical presentations

Fifteen (88.2%) patients presented with extremity swelling, eleven (64.7%) with extremity pain, and two (11.8%) with dyspnea. One (5.8%) patient had asymptomatic IVC filter-associated caval thrombus at the time of scheduled IVC filter removal.

### Thrombosed venous segments

Obstructed venous segments included the IVC and lower extremity (*n* = 9; 52.9%), isolated lower extremity (*n* = 4; 23.5%), IVC (*n* = 2; 11.8%), thoracic central veins and superior vena cava (*n* = 1; 5.9%), and isolated thoracic central vein (*n* = 1; 5.9%).

### Etiologies

Nine (52.9%) patients had obstruction secondary to malignancy, two (11.8%) had IVC filter-associated obstruction, two (11.8%) had occlusion of a previously placed venous stent, two (11.8%) had an unknown origin despite hematologic evaluation, and one (5.9%) had obstruction secondary to pacemaker leads. Malignancies included cervical cancer (*n* = 3; 17.6%), pancreatic adenocarcinoma (*n* = 1; 5.9%), large cell neuroendocrine carcinoma (*n* = 1; 5.9%), periampullary carcinoma (*n* = 1; 5.9%), sarcoma (*n* = 1; 5.9%), gastric adenocarcinoma (*n* = 1; 5.9%), and mucoepidermoid lung carcinoma (*n* = 1; 5.9%). Five (29.4%) patients had cardiopulmonary disease.

### Outcomes

Gender, age, presenting symptoms, procedural indications, obstructed venous segments, the Protrieve Sheath access and deployment sites, thrombectomy devices utilized, need for stent reconstruction, technical success, clinical success, adverse events removed thrombi analyses, and mortality were recorded. Technical success was defined as the deployment of the Protrieve Sheath funnel in the IVC with confirmation of wall-to-wall apposition on venography. Clinical success was described as resolution of presenting symptoms without new venous thromboembolic symptoms. Adverse events included the Protrieve Sheath access site complications and clinically significant thromboembolic events.

## Results

### Accesses

**Fig. 1:**
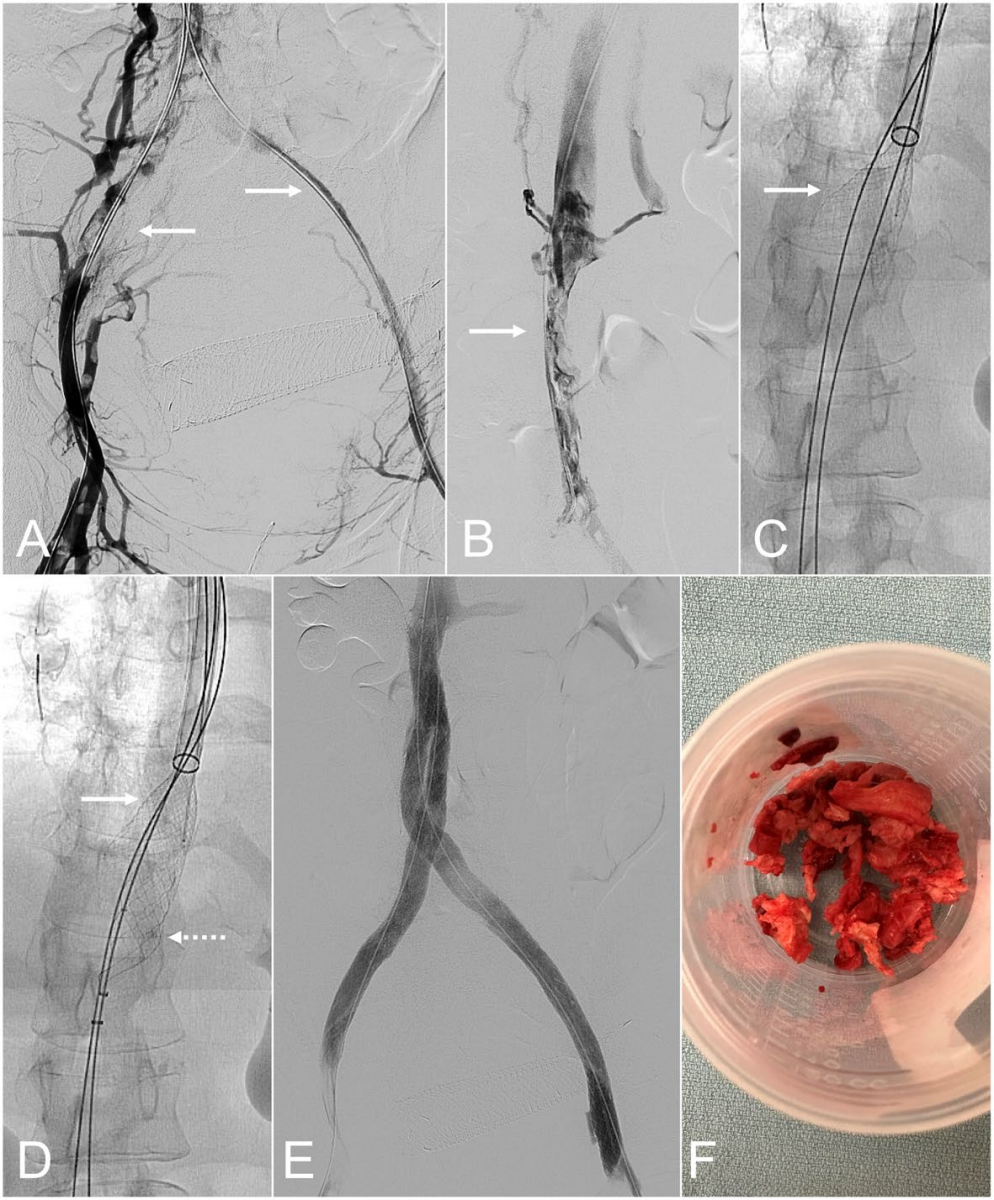
(Patient 1): *37-year-old female with metastatic cervical cancer with bilateral lower extremity swelling and pain*. **A** Bilateral lower extremity ascending venography demonstrated extrinsic compression and acute thrombus throughout both iliocaval venous segments (solid arrows). **B** Inferior vena cava venography showed acute thrombus throughout the intra-renal inferior vena cava (white arrow). **C** The Protrieve Sheath was placed and the funnel deployed in the intrahepatic inferior vena cava (solid arrow). **D** The ClotTriever System (dashed arrow) was then advanced into the Protrieve Sheath (solid arrow) and large-bore thrombectomy of both lower extremities was performed. Stent reconstruction of the inferior vena cava and bilateral iliocaval venous segments was then performed using Abre venous stents and Viabahn stent-grafts. **E** Completion bilateral lower extremity ascending venography demonstrated brisk in-line flow from both common femoral veins, through the stent reconstructions, to the right atrium. **F** Histologic thrombus analysis, from both the ClotTriever System and Protrieve Sheath, was consistent with cervical cancer

*Results are summarized in* Table 1. *Clinical Cases are shown in *Fig. 1. The Protrieve Sheath access sites included the right internal jugular (*n* = 15; 88.2%) and right femoral veins (*n* = 2; 11.8%). The Protrieve funnel deployment sites included intrahepatic IVC in 15 patients (*n* = 15; 88.2%) and inferior cavoatrial junction in two (*n* = 2; 11.8%).

### Interventions

All patients underwent venous thrombectomy after deployment of the Protrieve Sheath. Thrombectomy devices included: ClotTriever System (Inari Medical) (*n* = 15; 88.2%), Inthrill Thrombectomy System (Inari Medical) (*n* = 4; 23.5%), FlowTriever System (T20 and T24) (Inari Medical) (*n* = 2; 11.8%), Lightning Flash 16 Aspiration System (Penumbra; Salt Lake City, UT) (*n* = 2; 11.8%), Cleaner Rotational Thrombectomy System (Argon; Plano, TX) (*n* = 1; 5.9%), RevCore Thrombectomy System (Inari Medical) (*n* = 1; 5.9%). Ten (58.8%) patients underwent subsequent venous stent reconstruction of the involved venous segments given the significant residual thrombus burden and/or refractory stenosis resistant to angioplasty. Stent choices were per the operator's discretion. Total number of stents deployed was 21 with utilized stents including Abre (Medtronic; Minneapolis, MN) (*n* = 13; 61.9%), Venovo (Becton, Dickinson and Company; Franklin Lakes, NJ) (*n* = 4; 19.0%), Wallstent (Boston Scientific; Marlborough, MA) (*n* = 1; 4.8%), Gianturco Z-stent (Cook Medical; Bloomington, IN) (*n* = 1; 4.8%), and Viabahn stent-grafts (Gore Medical; Flagstaff, AZ) (*n* = 2; 9.5%). The average stent diameter was 15.3 ± 2.6 mm (range: 12–20 mm). Two IVC filters were removed including one Gunther Tulip (Cook Medical) and one Bard G2 (C. R. Bard, Inc; Murray Hill, NJ). Intravascular ultrasound was used in 15 (88.2%) patients.

### Outcomes

Technical success was achieved in all patients. Clinical success was achieved in 16 (94.1%) patients with improvement or resolution of the presenting symptoms and no development of new venous thromboembolic symptoms, such as extremity swelling/pain, chest pain, and dyspnea. One (5.9%) patient had persistent bilateral lower extremity swelling due to residual bulky and solid chronic thrombus that was resistant to thrombectomy. One (5.9%) patient had clinical resolution of swelling and pain but required additional thrombectomy twenty days later. No adverse events, including the Protrieve Sheath access site adverse events or clinically significant thromboembolic events, occurred.

Histologic thrombus analysis was performed in fifteen (88.2%) patients. Ten (66.7%) samples were benign and five (33.3%) were malignant thrombus. Malignant samples were all consistent with the known primary malignancy.

Nine (52.9%) patients expired during the study period.

## Discussion

In this study, 17 patients with various etiologies of deep venous obstruction underwent recanalization procedures with the Protrieve Sheath acting as a conduit for large-bore thrombectomy instruments while also providing protection from intraprocedural venous thromboembolic events.

Embolic events remain a longstanding procedural concern since the advent of endovascular techniques [9]. Arterial embolic events during carotid, renal, lower extremity, and coronary interventions are associated with significant morbidity and mortality [10–14]. Embolic protection devices have demonstrated reduction of intraprocedural thromboembolism events [15]. With the exception of flow reversal devices, embolic protection in the arterial space requires crossing of the thrombus prior to protection device deployment, which increases the risk of distal embolization. Given the different flow dynamics and quantity/characteristics of emboli in the veins, embolic protection in the deep venous system necessitates a novel design. IVC filters are contraindicated when using large-bore mechanical thrombectomy devices such as the ClotTriever System due to risk of entanglement.

The Protrieve Sheath utilizes a 33.5-mm diameter mesh funnel suitable for placement in the inferior vena cava with a low risk of injuring the caval wall. While blood can still flow through the funnel back to the heart, embolized materials are captured and aspirated utilizing a large-bore system. The Protrieve Sheath is a suitable alternative to temporary IVC filter placement or off-label use of FlowTriever disks for embolic protection during mechanical thrombectomy, complex IVC filter retrieval, or other large-bore venous cases with increased risk of venous embolism. The Protrieve Sheath can additionally serve as an access site for performing venography, recanalization, angioplasty, and stent placement, reducing the need for multiple venous punctures.

The risk of venous thromboembolism is heightened in patients with right-to-left cardiopulmonary shunts. Patients with atrial septal defects, ventricular septal defects, and arteriovenous fistulas are at increased risk for arterial ischemia in the setting of “paradoxical” thromboembolism [16]. Although advances in mechanical thrombectomy devices have increased the feasibility and safety of treating complex DVT, the risk of thrombus embolization and complications of PE or paradoxical embolization remain [4].

Complicated IVC filter removal poses a risk of filter fracture and component embolization with associated adverse outcomes [6, 17]. In the setting of infectious/malignant thrombi, there is an additional risk of seeding and spread of the disease process in the heart or lungs. In this cohort of patients, five (55.6%) of the nine with known malignancy demonstrated pathologic concurrence in the captured emboli corresponding to the primary malignancy. This confirmed intravascular tumor extension in these patients with potential therapeutic implications. In all seventeen patients, no new pulmonary emboli were seen on post-procedural imaging.

Limitations of the study include its retrospective nature with a small sample size. The benefit of the Protrieve Sheath over standard techniques is so far theoretical and anecdotal given the lack of comparative analysis. More studies are warranted.

## Conclusion

The Protrieve Sheath use during venous recanalization procedures is feasible with potential benefit of preventing clinically significant thromboembolism during large-bore instrumentation.

## Data Availability

Data for this study available upon reasonable request.

## Abbreviations

IVC: Inferior vena cava
DVT: Deep vein thrombosis
